# Completion of the Genome Sequence of a Historic CDV Vaccine Strain, Rockborn: Evolutionary and Epidemiologic Implications

**DOI:** 10.3390/vetsci13010081

**Published:** 2026-01-14

**Authors:** Zsófia Lanszki, Krisztián Bányai, Ágnes Bogdán, Gábor Kemenesi, Georgia Diakoudi, Gianvito Lanave, Francesco Pellegrini, Nicola Decaro, Vito Martella

**Affiliations:** 1 National Laboratory of Virology, Szentágothai Research Centre, University of Pécs, 7624 Pécs, Hungary; lanszkizsofi@gmail.com (Z.L.); kemenesi.gabor@gmail.com (G.K.); 2 Faculty of Sciences, Institute of Biology, University of Pécs, 7624 Pécs, Hungary; 3 Department of Medical Biology, Medical School, University of Pécs, 7624 Pécs, Hungary; bkrota@hotmail.com (K.B.); agnesbogdan@gmail.com (Á.B.); 4 Department of Pharmacology and Toxicology, University of Veterinary Medicine, 1078 Budapest, Hungary; 5 National Laboratory for Infectious Animal Diseases, Antimicrobial Resistance, Veterinary Public Health and Food Chain Safety, HUN-REN Veterinary Medical Research Institute, Hungária krt. 21., 1077 Budapest, Hungary; 6 Department of Veterinary Medicine, University of Bari Aldo Moro, 70010 Valenzano, Italy; georgia.diakoudi@uniba.it (G.D.); gianvito.lanave@uniba.it (G.L.); francesco.pellegrini@uniba.it (F.P.); nicola.decaro@uniba.it (N.D.)

**Keywords:** canine distemper, whole genome sequencing, phylogenetic analysis, recombination

## Abstract

The historic Rockborn strain of the canine distemper virus was widely used as a vaccine, but its use was discontinued due to safety concerns. Yet, Rockborn-like canine distemper virus strains are still used in some vaccine formulations. Genetic analysis of this strain was previously limited to the H gene, leaving its full evolutionary and pathogenic potential unclear. This study aimed to determine the complete genome sequence of the Rockborn strain to reconstruct its origin, understand its evolution, and provide a reference for improving diagnostics and future research on virulence markers. An amplicon-based sequencing protocol using MinION nanopore technology was employed to determine the complete genome of the Rockborn-46th laboratory strain. The genome was assembled, annotated, and analyzed in comparison with 223 genomes. The complete genome of the Rockborn strain was 15,690 nucleotides in length. Phylogenetic analysis revealed that Rockborn forms a unique lineage with field isolates from a masked civet in China and a dog in the United States. Crucially, a significant recombination event was identified, showing that the Rockborn strain acted as a parental strain, contributing its F and H genes to create mosaic viruses. The full-genome characterization of the Rockborn strain confirms that Rockborn-like viruses persist and actively contribute to the evolution of canine distemper virus through recombination. This finding highlights the inadequacy of single-gene analysis for diagnostics and surveillance, and underscores the necessity of whole-genome sequencing to accurately track the virus epidemiology and evolution.

## Simple Summary

Canine distemper is a serious, contagious disease in dogs and other animals, and vaccines are crucial for its control. An old vaccine strain, called Rockborn, was widely used but later withdrawn from many markets due to safety concerns, although vaccines with Rockborn-like viruses are still on the market. We sequenced the entire genome of a Rockborn virus cell passage available in our laboratory to understand its evolution and improve future diagnostic tools. Our analyses revealed that viruses similar to the Rockborn strain are still circulating in wildlife and domestic dogs. More importantly, it was discovered that the Rockborn strain has been exchanging genetic material with other canine distemper viruses, creating new, recombinant viruses.

## 1. Introduction

Canine distemper virus (CDV) is a paramyxovirus (*Paramyxoviridae* family, *Morbillivirus* genus) and possesses an enveloped virion with a minus-sense single-stranded RNA genome of ~15 kilobases in length. Among the six main primary gene products (N, P, M, F, H, and L) (Supplementary Table S1 [1,2,3,4,5,6,7,8,9,10,11,12]), the H gene is the most characterized genomic region. The H gene product acts as a cell attachment protein and mediates entry; moreover, antibodies against the H protein protect against infection and disease [13]. The exposure of the H protein to the immune system and the widespread use of CDV vaccines globally are thought to contribute to the genetic diversity among circulating strains. A genotyping classification scheme of CDV has been established based on the H gene [14,15,16,17].

The historic CDV strain, Rockborn, was isolated and attenuated by serial passage in the late 1950s [18]. Rockborn strain-based vaccines have been commercialized and distributed globally since the 1960s. This vaccine was shown to elicit a strong immune response and protect against disease caused by field CDV strains. However, safety concerns started to rise in the 1970s due to observed residual virulence. Although the etiological role was never fully proven, cases of vaccine-related encephalitis in the United States in the mid-1990s led to the definitive withdrawal of Rockborn-based CDV vaccines from the market [19,20]. Yet, several modern vaccines seemingly contain Rockborn-like viruses [21].

Overall, the high genetic identity between vaccine stock/laboratory strains and field isolates of vaccine-origin clinical cases was consistent with the hypothesis that the vaccine viruses retained residual virulence, likely in co-infection with other immunosuppressive pathogens. This was attributed to an intrinsic virulence of low-passage stocks established on primary dog kidney (DK) cells and grown for vaccine passage cultures on Madin Darby Canine Kidney (MDCK) cells. In most cases, increased virulence of the dog-kidney-cell-attenuated CDV was suspected to have been triggered by the contemporaneous presence in the vaccine formulations of live modified canine adenovirus type-1 [21]. Under experimental conditions, re-acquisition of virulence by the kidney-cell-attenuated Rockborn virus has been demonstrated in vivo after six sequential passages in dogs and in vitro after ten passages in primary dog lung macrophages, but the molecular bases of this phenotypic change were not investigated [22].

So far, genetic analysis of the Rockborn strain has been limited to the H gene. On sequence analysis of the full-length H gene of a cell passage (46th) of strain Rockborn, the virus and related strains were found to share 92–97% nucleotide (nt) and 91–96% amino acid (aa) sequence homology to other lineages of CDV. Additional partial H gene sequences were available for comparison, originating from vaccine-associated clinical cases and wild animals. Sequence divergence among strains varied slightly, with both nt and aa identities exceeding 99% [21]. With the determination of the full genome, we had the following objectives: (i) to better reconstruct the origin and evolution of the Rockborn strain and (ii) to extend the information with implications for diagnostic developments. Additionally, the reference genome sequence presented in this study will help identify virulence markers in clinically relevant Rockborn-associated CNS infections, if any.

## 2. Materials and Methods

### 2.1. Sequencing

The complete genome sequence of Rockborn-46th was determined using an amplicon-based sequencing protocol that combines amplification of adjacent CDV genomic fragments with MinION nanopore sequencing (Oxford Nanopore Technologies, Oxford, UK). The amplicon-based sequencing method for canine distemper virus, together with the primers employed, has been described previously [23], and the complete protocol is also available on the protocols.io page (https://www.protocols.io/view/universal-amplicon-based-sequencing-method-for-can-x54v9j6mpg3e/v1/metadata (accessed on 24 November 2021)). In brief, cDNA was prepared from extracted viral RNA with Superscript IV (Invitrogen, Carlsbad, CA, USA) using random hexamers. Overlapping PCR products were amplified from the cDNA with the Q5 Hot Start HF Polymerase (New England Biolabs, Ipswich, MA, USA) with multiple primer sets in parallel pools and then purified with AMPure XP beads (Beckman Coulter, Brea, CA, USA). The primer sequences used were designed to generate overlapping amplicons of approximately 1000 and 2000 nt in size and no modifications were made to the original CDV sequencing protocol. The end-repair and dA tailing were performed with the NEBNext Ultra II End Repair/dA-Tailing Module (New England Biolabs, USA). DNA barcodes EXP-NBD196, (Oxford Nanopore Technologies, UK) were ligated with NEBNext Ultra II Ligation Module (New England Biolabs, USA). After purification with Ampure XP beads, the AMII sequencing adapters were ligated with NEBNext Quick Ligation Module. Sixty ng of the final library was loaded onto a R9.4.1 (FLO-MIN106D) flow cell.

Also, consensus primers were designed to obtain the sequence of the 5′ (p2679 5′-ACCAGAMAAAGTTGGCTAWGGATAGW-3′) and 3′ genome (p2683R 5′-ACCAGACAAAGCTGGGTATGATAACT-3′) terminations.

### 2.2. Genome Assembly and Annotation

Base-calling and barcode demultiplexing were performed with Dorado version 1.1.0. Sequence reads below the expected size were removed and the consensus sequence was assembled by mapping to the MN267060 using the Geneious mapper (version Geneious Prime 2021.6.0.). Medaka (version 2022.1.1.) was used to map trimmed reads against a preliminary consensus to generate polished consensus sequences. The generated consensus sequences were manually checked for base-calling errors, especially in the homopolymeric regions. The genome was annotated using the ORF Finder (https://www.ncbi.nlm.nih.gov/orffinder/ (accessed on 5 September 2025)).

### 2.3. Phylogenetic Analysis

Sequences for the N, P, M, F, H, and L genes were first aligned using the MAFFT webserver with default parameters (https://www.ebi.ac.uk/jdispatcher/msa/mafft (accessed on 6 September 2025)). Thereafter, the IQ-TREE webserver (http://iqtree.cibiv.univie.ac.at/ (accessed on 6 September 2025)) was used to select substitution models and reconstruct maximum-likelihood phylogenetic trees, with 1000 ultrafast bootstrap replicates. The phylogenetic analyses were conducted under the GTR + F + I + G4 substitution model, which was identified as the best-fitting model according to the Akaike Information Criterion (AIC). Subsequently, the resulting trees were edited in the iTOL webserver (https://itol.embl.de/ (accessed on 7 September 2025)).

### 2.4. Recombination Detection

Whole-genome sequences were used to identify potential recombination events in Rockborn using the algorithms implemented in the Recombination Detection Program v.4.101 (RDP4) [24]. Default settings were used for each algorithm. A recombination event was accepted when detected by 7 distinct methods (RDP, GENECONV, BootScan, MaxChi, Chimaera, SiScan, 3Seq) implemented in the program, each with a *p*-value < 5 × 10^−4^.

### 2.5. Sequence Deposition

The genome sequence of the Rockborn strain was deposited in GenBank under the accession number PX254110.

## 3. Results

A total of 551.860 reads were mapped to CDV. The mean vertical coverage was about 44,000×. The assembled genome was 15,690 nt in length, with 107 and 105 nt long UTRs at the 3′ and 5′ ends, respectively. The lengths of N, P, M, F, H and L genes (and their respective proteins) were 1572 nt (523 aa), 1524 nt (507 aa), 1008 nt (335 aa), 1989 nt (662 aa), 1824 nt (607 aa) and 6555 nt (2184 aa), respectively. The genome and gene-wise sequence homologies showed values of over 98% and 99%, respectively, to the most closely related CDV strains, many, if not all, of which are putative Rockborn-derived vaccines and wild-type strains (Table 1).

**Figure 1:**
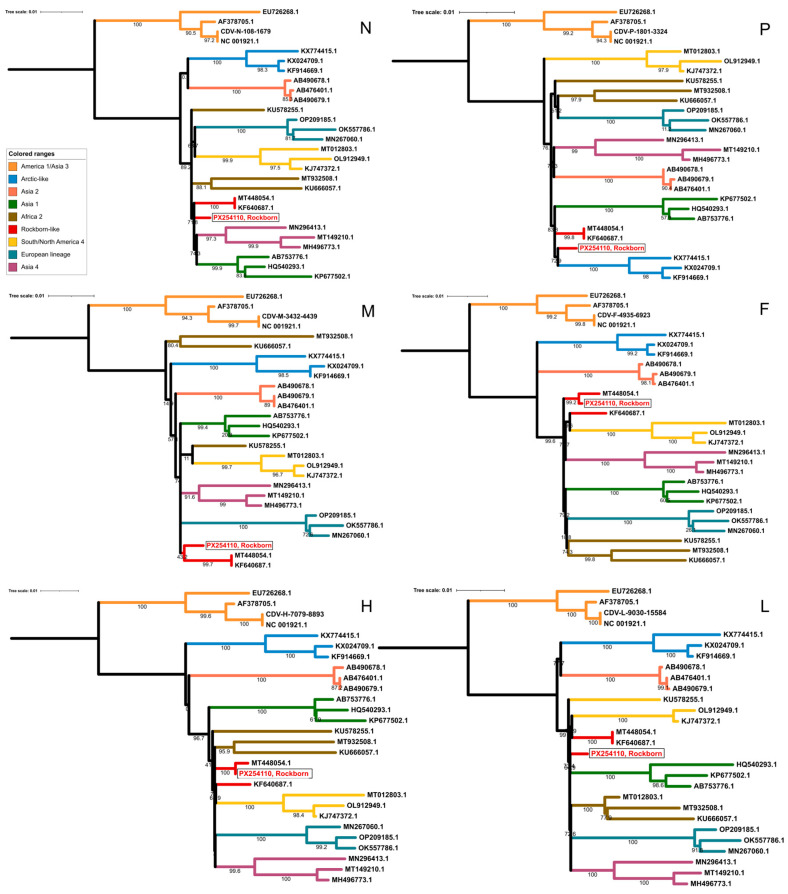
Gene-wise maximum-likelihood phylogenies based on 28 CDV sequences for the N, P, M, F, H, and L genes. Major CDV lineages are color-coded, and the Rockborn strain is highlighted in red and boxed. The clustering patterns of the Rockborn strain in the F and H genes are indicative of a recombination event. Bootstrap support values are shown at key nodes.

Next, whole-genome and gene-wise phylogenies were carried out (Figure 1). A whole-genome-based alignment was assembled from 223 reference genome sequences available in the GenBank and the newly determined Rockborn sequence. After a preliminary analysis, 27 relevant sequences were retained for further processing; these sequences represented the main genetic clades. The genome-wise phylogeny indicated that strain Rockborn clusters with two field strains for which full-length genomes are available, the HN19 strain from a masked civet in China and the R252 strain from a dog in the United States. These features indicate that strain Rockborn, along with some recent field isolates, constitutes a unique genetic lineage alongside more deeply analyzed CDV lineages. A closer look revealed that R252 and HN19 CDVs share greater genetic relatedness across the entire genome, except for the region spanning the F and H genes, from nt positions 4935 to 6923 and 7079 to 8902, respectively, in the masked civet-origin CDV strain (GenBank ID: MT448054). In this data set, analysis of this genomic region showed that the masked civet-origin HN19 strain was more closely related to Rockborn than to the canine-origin R252 strain.

**Figure 2:**
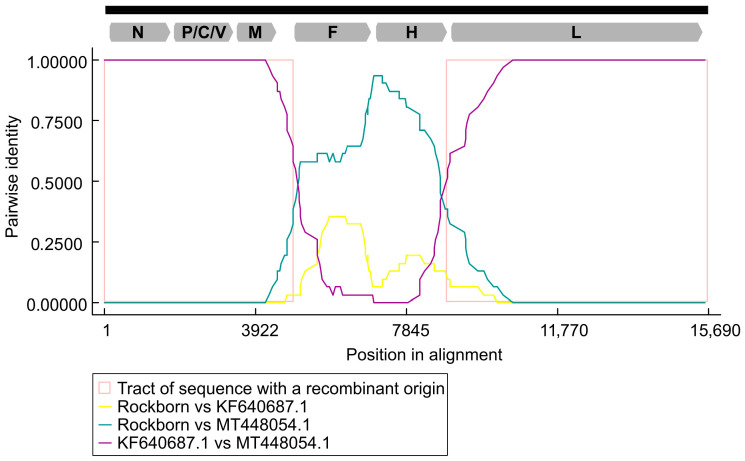
Corrected pairwise identity analysis generated using the RDP4 software (version 4.4). Pairwise identity plot obtained by comparing the Rockborn-46th strain with two closely related CDV genomes, HN19 (MT448054.1) and R252 (KF640687.1), showing identity values across the full genome alignment and the corresponding breakpoint confidence intervals. Different colors are used to depict the different identity plots. The genome structure is also shown.

These findings suggest that past recombination events led to the mosaic structure of the genomes of some strains within the Rockborn lineage. This peculiar pattern of gene-wise relationship among Rockborn-like strains with available full-length genomes was confirmed by recombination analysis using dedicated bioinformatic analyses (Figure 2). We used the whole alignment containing 224 sequences as input to run the analysis with default parameters. As a result, an intra-lineage recombination event was observed within Rockborn-like strains (all but one algorithm showed evidence of recombination), placing the breakpoint sites at nt 4934 and 8902 (99% CI, 4747–5036 and 8482–8974, respectively). This region spans the entire F and H genes and may include short fragments of non-coding regions upstream and downstream, as indicated by the 99% CI values.

## 4. Discussion

This study presents the complete genome sequence of the historic CDV strain Rockborn, revealing its ongoing relevance in the contemporary epidemiological landscape. These new data not only resolve long-standing questions about the genetic makeup of this lineage beyond the H gene but also underscore the complex evolutionary forces, particularly recombination, that shape CDV diversity [17,21].

Given the official withdrawal of Rockborn-based vaccines from many markets in the mid-1990s due to safety concerns [19,20], a significant aspect of this whole-genome sequencing study is the confirmation that Rockborn-like viruses or Rockborn-derived recombinants continue to circulate in dogs and wildlife. Based on the H gene sequence, Martella and co-workers identified two vaccines on the market containing Rockborn-like strains and noted that several field isolates from Austria and Japan showed nearly 100% nucleotide identity to the Rockborn-46th laboratory strain [21]. More recent studies have continued to detect these strains, reinforcing the idea of their natural persistence within and among different animal populations. Based on whole-genome sequence data generated in our study and database interrogation, we obtained additional evidence for the presence of Rockborn-like CDV strains in dogs and other carnivores (Table 1). Rockborn-like CDV was identified in a captive vaccinated fennec fox in Japan, 2017 [25], in non-vaccinated farmed masked civets in China, 2019 [26]. Also, Rockborn-like CDV was reported repeatedly, although sporadically, in domestic dogs in Brazil, 2015 [27], Canada, 2025 [28], and New Zealand, 2021–2024 [29], suggesting a continual spill-over of vaccine-derived Rockborn-like CDV strains into the environment.

The central finding of our genomic analysis was the identification of a clear recombination event, positioning the Rockborn strain as a probable parental strain to a mosaic virus circulating in the wild. While recombination in negative-sense RNA viruses was once considered a rare phenomenon [30], a growing body of evidence now establishes it as a key force driving the evolution and genetic diversity of CDV [17,31,32]. Previous studies have extensively documented recombination events involving various CDV genotypes. Budaszewski and co-workers identified eight putative recombinant viruses, demonstrating that homologous recombination is a frequent event in natural CDV populations and that vaccine strains, particularly from the America-1 lineage, play a significant role in shaping viral evolution [32]. Similarly, Yuan et al. [31] concluded that recombination is a key evolutionary force after identifying six distinct events and noting that viruses isolated from different host species—such as minks, seals, and raccoons—can recombine, contributing to the broad host range and adaptability of CDVs. Our study demonstrates that the historic Rockborn lineage is actively participating in this process, dispersing genome fragments of the original strain into other CDV strains over the long term via recombination. The mosaic genome structure observed, with parental contributions from strains infecting distantly related hosts like masked civets and domestic dogs, exemplifies the cross-species nature of these evolutionary events. In this case, the masked civet strain HN19 was closely related to the canine strain R252 across the genome, but the F and H genes, which were likely derived from strain Rockborn. These genes encode key proteins involved in cell-to-cell viral spread (F protein) and receptor interactions (H protein) [7,8,9,10,11]. Understanding the effects of the exchange of cognate genome regions is challenging and requires multiple studies using reverse genetics, in vitro or in vivo experiments, or both, for functional validation. Also, sampling bias and the heavy reliance on publicly available GenBank sequences might affect our analysis or the findings of analogous studies.

This work strongly emphasizes the limitations of relying on single-gene analyses for CDV genotyping and diagnostics. The H gene has traditionally been a target of phylogenetic studies due to its high variability and its role in immunity. Consequently, many diagnostic assays are designed to differentiate wild-type and vaccine strains based on specific polymorphisms within the H gene, or alternatively, other genomic regions [16,33,34,35,36,37,38]. However, the occurrence of recombination means that a virus can possess a gene from one lineage/sub-lineage while other parts of its genome originate from another lineage/sub-lineage. For example, the recombination event identified in this study appeared to affect the F and H genes, suggesting that any diagnostic or phylogenetic analysis based solely on the H gene would have masked and mischaracterized the recombinant virus. This risk has been pointed out in other whole-genome studies. Budaszewski and co-workers emphasized that genotyping based on partial genomic sequences could lead to misidentification and that complete genome analysis is necessary to detect recombination hot spots, which they frequently found in the F and H genes [32]. Likewise, Yuan and co-workers identified recombination events primarily in the P and L genes, regions often overlooked in standard genotyping [31]. Therefore, relying on single-gene assays in a landscape shaped by recombination can lead to flawed epidemiological conclusions and ineffective control strategies.

Vaccine-related disease has been reported on several occasions in dogs, but sporadically. In most cases, however, it has been difficult to establish whether the disease was induced by the vaccine virus or by field viruses infecting pups shortly before or after vaccine administration. This issue could not be ruled out definitively in earlier studies, as appropriate diagnostic tools were not available. Using a genotyping PCR assay that distinguishes between locally relevant CDV genotypes and vaccine viruses, all suspected cases of CDV vaccine-induced illness or death analyzed during a 2-year surveillance in Italy were caused by CDV field strains [16], suggesting that vaccine-induced residual virulence is rare. Optimized diagnostic tools capable of distinguishing between field and vaccine CDV strains could help better assess the magnitude of this phenomenon.

## 5. Conclusions

In conclusion, the full-genome characterization of the Rockborn strain not only fills a critical gap in our understanding of this historic lineage but also reveals its ongoing role in shaping the complex evolutionary dynamics of CDV through recombination. This underscores the need to shift towards whole-genome approaches for effective surveillance, accurate diagnostics, and a comprehensive understanding of CDV epidemiology and evolution.

## Figures and Tables

**Table 1:** CDV strains with the greatest sequence homology in the GenBank to strain Rockborn. Strains with uncertain metadata are indicated by *.

Gene	GenBank Entry, nt Identity	Origin		
		Host Species	Geographic	Year
Whole genome	MT448054, 98.8%	Masked civet	China	2019
	KF640687, 98.5%	Dog	United States	1970s *
	AF164967, 98.4%	Dog	United States *	1975 *
	EU716337, 98.2%	Dog	United States	2004
N	EU072200, 99.5%	vaccine	-	2006
	AY649446, 99.1%	Raccoon	United States	2001
P	EU072201, 99.7%	vaccine	-	2006
	KF640687, 99.1%	Dog	United States	1970s *
	MT448054, 99.1%	Masked civet	China	2019
M	EU072199, 99.7%	vaccine	-	2006
F	KY057355, 99.8%	Dog	Brazil	2015
	EU072198, 99.3%	vaccine	-	2006
	MT448054, 99.3%	Masked civet	China	2019
H	GU810819, 99.9%	Rockborn, 46th cell passage	-	2011
	LC498611, 99.8%	Fennec fox	Japan	2017
	GU266280, 99.8%	vaccine	-	2011
	FJ705238, 99.8%	vaccine	-	2010
	EF095750, 99.8%	vaccine	-	2010
	FJ461702, 99.7%	vaccine	-	2008
	MT448054, 99.6%	Masked civet	China	2019
	AF178039, 99.5%	Lesser panda	China	1999
	OQ282897, 99.8%	Dog	China	2021
	OQ282900, 99.7%	Dog	China	2021
	OQ282902, 99.6%	Dog	China	2021
	JX912968, 99.3%	Dog	Brazil	2008

## Data Availability

The data presented in this study are openly available. The genome sequence of the Rockborn strain was deposited in the Genbank (accession number, PX254110).

## Supplementary Materials

The following supporting information can be downloaded at: https://www.mdpi.com/article/10.3390/vetsci13010081/s1, Table S1: Functions of canine distemper virus genes.
