# Supplementary material for: Completion of the Genome Sequence of a Historic CDV Vaccine Strain, Rockborn: Evolutionary and Epidemiologic Implications
Source: Vet Sci. 2026 Jan 14;13(1):81. doi: 10.3390/vetsci13010081 (PMC12846550; doi:10.3390/vetsci13010081)
Supplement: Supplementary file 1 [file vetsci-13-00081-s001.zip › vetsci-3990982-supplementary.pdf]

Table S1. Function of canine distemper virus genes. The length of each gene (in brackets) is referred to the Rockborn genome sequence. For each gene, the highest nucleotide (nt) sequence identities (id) with 100% coverage, obtained by interrogating the GenBank database with Blastn (https://blast.ncbi.nlm.nih.gov/Blast.cgi), are reported. Vaccine sequences were excluded. The accession number and strain designation are reported. The references (Ref.) are also indicated.

Gene/protein	Function	Ref.	Blastn hit	Accession	Nt id %
N gene (1572 nt)	Encapsidation.<br>Delay of interferon response.	[1]	01-2689<br>A75/17	AY649446<br>AF164967	99.17<br>98.98%
P gene (1655 nt)	The phosphoprotein gene of the paramyxoviruses encodes multiple protein products (P, V and C) via transcriptional slippage/editing.		CDV-HN19<br>R252 Ohio	MT448054<br>KF640687	99.09<br>99.09
P protein	Primary translation product. Interaction with L protein and N-RNA template during transcription.	[2]			
V protein	Inhibition of STAT1- and STAT2 -mediated Type I Interferon signaling.	[3]			
C protein	Regulates viral replication, preventing "runaway" synthesis and dsRNA accumulation and delaying interferon activation.	[4]			
M (1008 nt)	Regulation of viral assembly and budding.	[5]	A75/17<br>01-2689	AF164967<br>AY649446	98.81<br>98.71
M-F intergenic region (about 400 nt)	Modulates virulence by controlling F protein expression levels.	[6]			
F gene (1989 nt)	Virus entry and cell-to-cell spread. Fusion of the viral envelope with cell membrane.	[7,8]	BRA/UEL-FRD/15<br>CDV-HN19	KY057355<br>MT448054	99.80<br>99.23
H gene (1824 nt)	Interaction with cell receptors<br>SLAM (CD150), Nectin-4 (PVRL4) and GliaR	[9–11]	CDV-HN19<br>Lesser panda	MT448054<br>AF178039	99.49<br>99.49
L gene (6555 nt)	Large (L) polymerase protein.<br>RNA dependent RNA polymerase, mRNA capping and methylation).	[12]	CDV-HN19<br>R252 Ohio	MT448054<br>KF640687	98.77<br>98.77
